# Cognitive-motor interference during walking with modified leg mechanics: a dual-task walking study

**DOI:** 10.3389/fpsyg.2024.1375029

**Published:** 2024-04-18

**Authors:** Norman Riedel, Michael Herzog, Thorsten Stein, Barbara Deml

**Affiliations:** ^1^Institute of Human and Industrial Engineering, Karlsruhe Institute of Technology, Karlsruhe, Germany; ^2^BioMotion Center, Institute of Sports and Sports Science, Karlsruhe Institute of Technology, Karlsruhe, Germany

**Keywords:** cognitive-motor interference, dual-task walking, lower extremity loading, modified leg mechanics, exoskeleton, attentional processes, motor control

## Abstract

**Background:**

The use of mobile exoskeletons as assistive walking devices has the potential to affect the biomechanics of the musculoskeletal system due to their weight and restricted range of motion. This may result in physical and cognitive load for the user. Understanding how lower extremity loading affects cognitive-motor interference is crucial for the design of wearable devices, including powered exoskeletons, and the development of effective training interventions.

**Objective:**

This study aims to examine the effects of modified leg mechanics on cognitive-motor interference in dual-task walking. Gait variability, as an indicator of motor control, was analyzed to investigate its relation to cognitive task difficulty and to determine whether lower extremity loading modifies this relationship. Additionally, the impact on the gait pattern, as represented by the mean values of spatio-temporal gait parameters were investigated.

**Method:**

Fifteen healthy young adults walked on a treadmill with and without weight cuffs bilaterally attached to their thighs and shanks while performing a visual-verbal Stroop test (simple task) and a serial subtraction task (difficult task). Dependent variables include mean values and variability (coefficients of variation) of step length, step width, stride time and double support time. Additionally, secondary task performance as correct response rates and perceived workload were assessed.

**Results:**

Double support time variability decreased during dual-task walking, but not during walking with modified leg mechanics while performing the difficult secondary task. Walking with modified leg mechanics resulted in increased gait variability compared to normal walking, regardless of cognitive load. During walking with modified leg mechanics, step length, step width, and stride time increased, while double support time decreased. The secondary tasks did not affect the gait pattern.

**Conclusion:**

The interplay between an external focus of attention and competition for attentional resources may influence the variability of double support time. The findings suggest that walking with modified leg mechanics could increase cognitive-motor interference for healthy young adults in demanding dual-task situations. Therefore, it is important to analyze the underlying mechanisms of cognitive-motor interference in the context of human-exoskeleton interaction.

## 1 Introduction

In daily life, we frequently engage in multiple tasks simultaneously. This can lead to impairments when cognitive resources are insufficient to handle these tasks at the same time. In situations involving both motor and cognitive tasks, this impairment is referred to as cognitive-motor interference. The analysis of cognitive-motor interference using a dual-task walking approach is widely used in clinical and epidemiological research (as reviewed in Al-Yahya et al., 2011; Bayot et al., 2018). This approach has proven effective in revealing aging effects (as reviewed in Beurskens and Bock, 2012) and effects due to neurodegenerative diseases such as Parkinson’s disease (as reviewed in Kelly et al., 2012) on cognitive and motor performance. However, this approach could also be useful to evaluate human-exoskeleton interaction. In this context, studies have either focused on biomechanical and physiological effects (as reviewed in Pinto-Fernandez et al., 2020) or cognitive effects (Bequette et al., 2020; Upasani and Srinivasan, 2023), but not the combined effects, i.e., the cognitive-motor interference.

According to Yogev-Seligmann et al. (2012), two main factors determine the extent of interference and prioritization of tasks in dual-task walking. The first factor *postural reserve* includes all individual aspects that ensure postural control and reflects the “capability to respond most effectively to a postural threat” (Yogev-Seligmann et al., 2012, p. 766). The second factor *hazard estimation* refers to the cognitive capability of self-awareness including the estimation of environmental hazards and self-limitations. Young healthy adults are assumed to have an intact postural reserve and hazard estimation, which allows them to concentrate on performing secondary tasks without impairing their gait performance. However, studies have also shown that interference in dual-task walking can also be observed in healthy young subjects (Patel et al., 2014; Wrightson and Smeeton, 2017; Hamacher et al., 2019b). External factors, such as complex environmental influences or challenging motor tasks can demand the postural reserve (Yogev-Seligmann et al., 2012). This can result in a larger proportion of conscious attention being allocated to the motor task to maintain a stable gait. Kao and Pierro (2022) investigated cognitive-motor interference during walking with and without continuous treadmill platform sways and found that participants prioritized the walking task during the perturbed walking condition. Reiser et al. (2019) found a decrease in the parietal P3 amplitude with increasing movement complexity in an outdoor environment using mobile electroencephalography. This suggests that there is a higher cognitive workload associated with increasing movement complexity.

In the field of dual-task studies, examining gait parameters provides insight into the complex relationship between motor performance and cognitive demands. Al-Yahya et al. (2011) conducted a meta-analysis that highlighted the effects of dual-tasking on various mean and variability gait parameters. Gait variability is considered an indicator of motor control (Newell and Corcos, 1993; Hausdorff, 2005). Low variability suggests reliance on automatic processes, whereas high variability signifies the engagement of attentional resources in motor control. Studies have linked high gait variability to negative health outcomes, including falls in older adults and various comorbidities (Pieruccini-Faria et al., 2020). According to Tian et al. (2017), temporal and spatial gait variability parameters may be associated with brain areas related to sensorimotor integration and coordination in older adults. The addition of cognitively demanding secondary tasks or physical perturbations that engage the same brain areas can cause interferences that lead to changes in gait variability. While some studies report an increase in gait variability among healthy older adults when faced with a secondary task (as reviewed in Smith et al., 2017), others demonstrate a decrease (Lövdén et al., 2008; Decker et al., 2016; Hamacher et al., 2019a). This divergence in findings is attributed to a shift in attentional focus from walking to the secondary task, facilitating more automated walking patterns. The Dual-Process Model, initially proposed by Huxhold et al. (2006) in the context of standing balance control, provides a framework for understanding these observations. According to this model, simple cognitive tasks promote an external focus of attention, thereby enhancing automated motion execution and reducing variability. Conversely, complex cognitive tasks lead to competition for attentional resources, outweighing the benefits of an external focus and resulting in increased variability. Thus, a U-shaped relationship emerges between gait variability and cognitive task difficulty, with variability being high during single-task walking, decreasing with a simple secondary task, and increasing again with a complex secondary task.

Considering that factors such as physical effort, modified biomechanical structures and the use of assistive devices are supposed to increase the use of attention-demanding cognitive resources (Clark, 2015), the dual-task walking approach and the Dual-Process Model can provide valuable insights in the context of human-exoskeleton interaction. Findings are important for evaluating the current state of cognitive and motor adaptation to a system, as well as to develop and evaluate appropriate training interventions. In a field study with participants completing an obstacle course, Bequette et al. (2020) reported slowed reaction times in a visual search task for some participants when wearing a powered lower limb exoskeleton. The overall perceived workload assessed with the NASA-TLX was significantly higher in powered and unpowered walking compared to walking without the exoskeleton. Riedel et al. (2023) investigated effects of modified leg mechanics using weight cuffs attached to both upper and lower legs on cognitive performance and perceived workload during dual-task walking on a treadmill. Participants who started with the loaded walking condition showed significant performance decrements on a subtraction task during loaded but not during unloaded walking. Consistent with Bequette et al. (2020), physical and mental demand assessed with the NASA-TLX increased during loaded walking, however not significantly for mental demand.

This study aims to examine the effects of modified leg mechanics on cognitive-motor interference in dual-task walking. Riedel et al. (2023) initially analyzed subjective measures and behavioral parameters. However, motion data can also provide valuable information regarding cognitive-motor interference. The present paper analyzed gait variability, as an indicator of motor control, to investigate its relation to cognitive task difficulty and to determine whether lower extremity loading modifies this relationship. Additionally, the impact on the gait pattern, as represented by the mean values of spatio-temporal gait parameters was investigated. Weight cuffs attached bilaterally to the thighs and shanks manipulated the biomechanics of the musculoskeletal system and added complexity to the motor task. According to the Dual-Process Model, it was hypothesized that loaded walking would exhibit a U-shaped relationship between gait variability and cognitive task difficulty, unlike unloaded walking (H1a). We predicted that gait variability would be higher during loaded walking compared to unloaded walking (H1b). Furthermore, changes were anticipated in the overall gait pattern, as indicated by mean spatio-temporal gait parameters, due to the weight cuffs (H2a) and the performance of secondary tasks (H2b).

## 2 Materials and methods

For the analysis of motor performance under different single and dual-task walking situations, a 2 × 3 within-subject experimental design was employed with two *Walking Conditions* (unloaded walking, loaded walking) and three *Task Conditions* (no secondary task, visual-verbal Stroop test, serial subtraction task).

### 2.1 Participants

Sixteen healthy young adults participated in the study. Only 15 participants (age: 24.3 ± 3.5 years; stature: 1.73 ± 0.09 m; body mass: 66 ± 10.1 kg; physical activity: 3.2 ± 0.3 days/week and 183 ± 25 min/week) were used for the analysis due to incomplete recording of motion data for one individual. The participants, consisting of eight females and seven males, were selected from the student population of the Karlsruhe Institute of Technology. Individuals with red-green visual impairment were excluded from the study. This research complied with the American Psychological Association Code of Ethics and was approved by the Ethics Committee of the Karlsruhe Institute of Technology. All participants provided written informed consent prior to participation.

### 2.2 Apparatus

**Figure 1:**
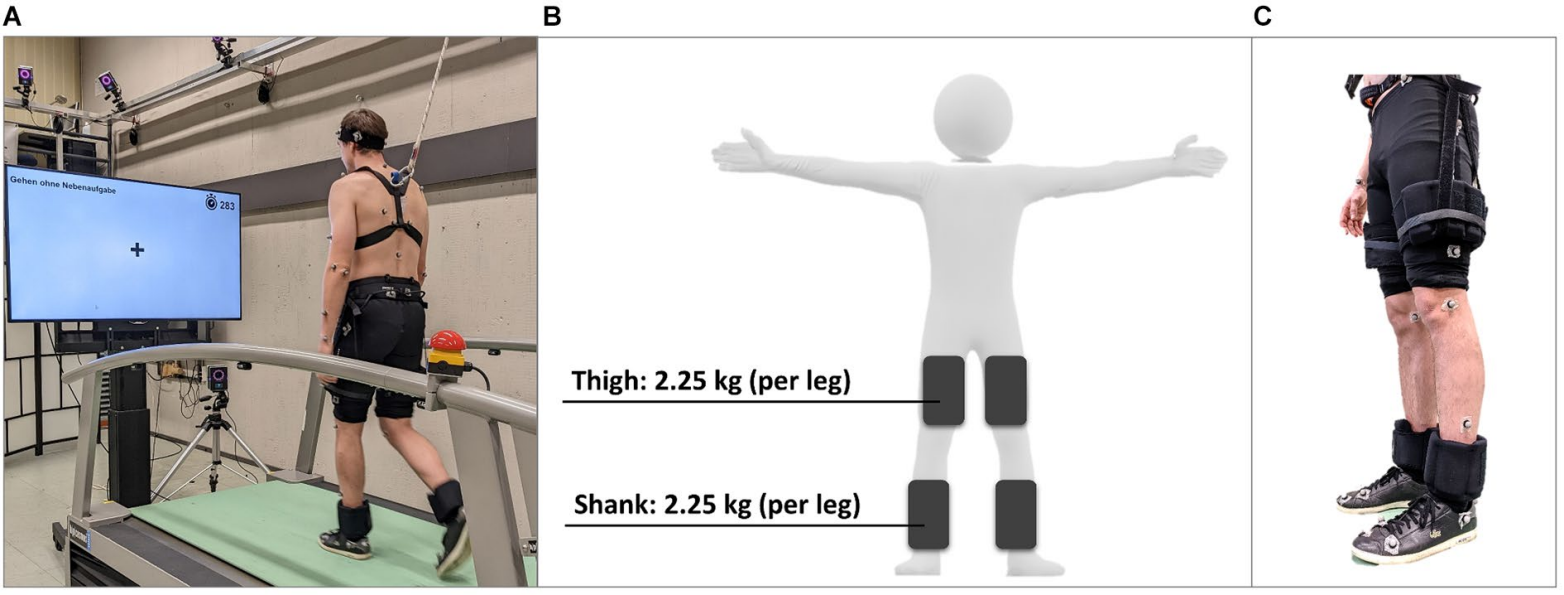
**(A)** Experimental setup. **(B)** Schematic representation of the positions of the weight cuffs. **(C)** Representation of the attachment of the weight cuffs to a participant. Image adapted from Riedel et al. (2023).

Participants walked on a treadmill (h/p/cosmos Saturn; Nussdorf-Traunstein, Germany) with and without weight cuffs bilaterally attached to the thigh and shank (see Figure 1). The total weight of the four weight cuffs was 9 kg (2.25 kg each), which is typical for lower-limb, gait-assisting exoskeletons (Bortole et al., 2013). A custom hip belt was designed to secure the weight cuffs to the thigh. The hip belt was a climbing harness without the leg elements. It consisted of a padded hip belt with side loops to which two Velcro straps were attached on each side. The weight cuffs were hooked onto the Velcro straps to prevent them from slipping down during movement. The Velcro straps could be adjusted in height, ensuring that the lower edge of the weight cuffs was positioned 10 cm above each participant’s knee joint axis. The weight of powered exoskeletons is strongly centered on the motors, which are located at the joints. A position close to the knee joint axis was chosen, which also ensures that movement is not restricted. For safety, a harness was used to secure participants during treadmill walking. In front of the treadmill a 65-inch monitor was mounted at a distance of 240 cm from the participants. The top edge of the monitor was set at the eye level of each participant. The monitor was used to display the current task to be performed, including the presentation of the stimuli of the secondary tasks. Participants were instructed to keep their eyes at the monitor to ensure an upright posture. An infrared camera system (Vicon Motion Systems; Oxford Metrics Group, Oxford, UK) equipped with 16 cameras (200 Hz) was employed to capture whole-body movements, using a modified Master-Motor-Map marker-setup with 56 markers (Mandery et al., 2016).

### 2.3 Secondary tasks

A wide variety of cognitive tasks exists that assess different cognitive functions. Al-Yahya et al. (2011) established a classification based on the type of mental processes required to perform the task. In this study, two cognitive tasks from different classifications were used: a visual-verbal version of the Stroop test (STR) (Stroop, 1935) as a discrimination task involving response inhibition and a serial subtraction task (SUB) as a mental tracking task.

**Figure 2:**
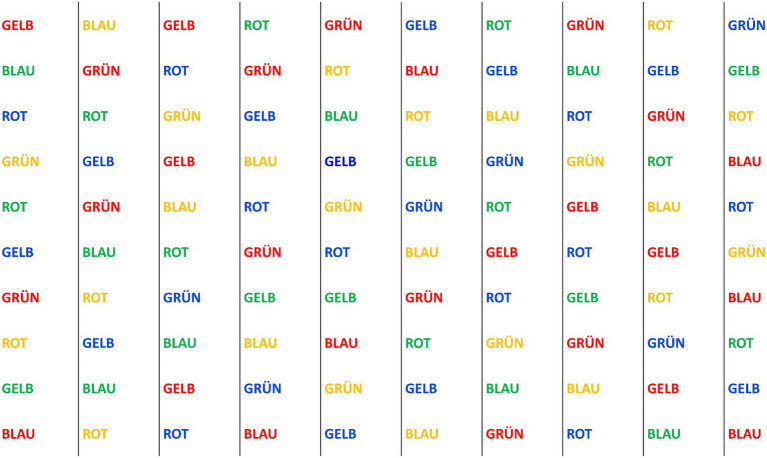
Example of stimulus used in the present study within the STR task.

STR involved the presentation of a 10 × 10 matrix containing color words (red, blue, green, and yellow) with incongruent word and color information, which was displayed on the monitor in front of the treadmill for a duration of 60 s (see Figure 2). To avoid learning effects, there were five different matrices, which were presented in random order. Participants were instructed to name the respective font color of the words as quickly as possible and without error. Participants started in the left top corner and continued column wise to the right. During SUB, a random three-digit number ranging from 201 to 999 was presented to the participants. They were then instructed to perform serial subtractions of seven from the presented number continuously for 60 s.

Riedel et al. (2023) reported the average score of the mental demand subscale of the NASA-TLX during unloaded single task walking (Score: 7), dual-task walking with STR (Score: 35) and dual-task walking with SUB (Score: 49). As these scores were significantly different from each other, STR is considered the simple secondary task and SUB is considered the difficult secondary task.

### 2.4 Procedure

For familiarization, participants first performed each secondary task for 80 s while seated. Afterwards they walked for 6 min on the treadmill according to recommendations by Meyer et al. (2019), before the preferred gait speed of 1.14 ± 0.08 m/s (4.1 ± 0.3 km/h) was determined using the method suggested by Jordan et al. (2007). The determined speed was maintained constant for all experimental conditions.

**Figure 3:**
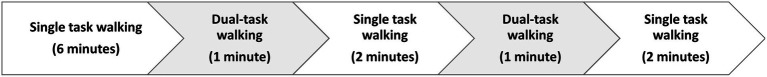
Schematic illustration of the protocol of a walking session. The same protocol was performed with and without weight cuffs.

The two walking sessions followed in a balanced order, each lasting 12 min in total. One session involved unloaded walking and one loaded walking. Figure 3 shows the protocol of a walking session. Both walking sessions started with a 6-min block of single task walking. This first block was intended to control adaptation processes, especially in the loaded walking session, and to ensure that subjects did not have to use cognitive resources to adapt to unfamiliar motor conditions. For example, adaptation to walking with unilateral attached weights is assumed to be completed after 45–50 strides (Noble and Prentice, 2006). This was followed by the first dual-task walking block for 1 min. To counteract cognitive fatigue, a 2-min block of single task walking followed before the second dual-task walking block was performed for 1 min. The sessions ended with another 2-min block of single task walking. The order of secondary tasks’ appearance has also been counterbalanced. No specific instructions were provided regarding which task to prioritize during dual-task walking. After the loaded walking session, an additional 18-min session followed, which was used to investigate (re-)adaptation processes. However, this session is not relevant for this paper. After the walking sessions, the secondary tasks were again performed while seated and served as control conditions.

### 2.5 Data processing and statistical analysis

The motion data was post-processed using Vicon Nexus 2.14.0 and Matlab R2023a (The MathWorks, Natick, MA, United States). The marker trajectories were smoothed in Matlab using a 6 Hz fourth order Butterworth low-pass filter (Gordon and Ferris, 2007). In each trial, the first and last 5 s were then cut off and the first 30 strides were extracted from the remaining section for the calculation of the mean values and coefficients of variation (CV) of step length, step width, stride time, and double support time. For single task walking, the last minute of the first 6-min block was considered for analysis. Segmentation of strides was performed according to Noble and Prentice (2006). The step length was determined as the anterior–posterior distance between the right and left heel markers at each heel contact. The step width was calculated using the medio-lateral distance between the right and left heel markers at each heel contact. The time between ipsilateral heel contacts represents the stride time, while the time at which both feet are in contact with the ground represents the double support time. The reliability of gait variability parameters is an ongoing discussion in the literature. Recommendations for the minimum number of strides to capture to reliably assess variability vary widely between six strides (Lord et al., 2011) up to more than a hundred strides (Hollman et al., 2010). Here, within-session reliability was assessed with the intraclass correlation coefficients (ICC) of all mean values and CV for single task walking (see Table 1). ICC values and their corresponding 95% confidence intervals were derived using a mean-rating (*k* = 3), absolute-agreement, 2-way mixed-effects model. Reliability values below 0.5 indicate poor reliability, values between 0.5 and 0.75 suggest moderate reliability, values between 0.75 and 0.9 indicate good reliability, while values greater than 0.90 indicate excellent reliability (Koo and Li, 2016).

**Table 1:** Intraclass correlation coefficients (ICC) of coefficients of variation (CV) and mean values of gait parameters.

	Loaded walking	Unloaded walking
**CV**		
Step length	0.866 [0.687, 0.951]	0.857 [0.658, 0.948]
Step width	0.933 [0.824, 0.976]	0.941 [0.823, 0.980]
Stride time	0.837 [0.618, 0.941]	0.675 [0.213, 0.883]
Double support time	0.924 [0.818, 0.972]	0.618 [0.153, 0.856]
**Mean values**		
Step length	0.978 [0.947, 0.992]	0.997 [0.993, 0.999]
Step width	0.983 [0.949, 0.994]	0.984 [0.962, 0.994]
Stride time	0.986 [0.966, 0.995]	0.995 [0.989, 0.998]
Double support time	0.992 [0.981, 0.997]	0.997 [0.992, 0.999]

ICC and their corresponding 95% confidence intervals were derived using a mean-rating (*k* = 3), absolute-agreement, 2-way mixed-effects model. Values represent ICC of gait parameters during single task walking. The 95% confidence intervals are represented in parentheses.

Data were tested for normal distribution using the Kolmogorov–Smirnov test. In contrast to the mean values, some CV showed a skewed, non-normal distribution. Since repeated measures ANOVAs (rmANOVA) are considered robust to violations of the normal distribution if the sphericity assumption is met (Schmider et al., 2010; Blanca et al., 2023) and transformations have considerable shortcomings (Feng et al., 2014; Blanca et al., 2017) parametric models were applied to the original data. The assumption of sphericity was tested with the Mauchly test, and in cases of violation, degrees of freedom were adjusted using the Greenhouse–Geisser correction. The significance level for all statistical analyses was set *a priori* at α = 0.05 and *post hoc* pairwise comparisons were Bonferroni corrected. Effect sizes are reported as partial eta squares ( $\eta_{p}^{2}$ ). Values between 0.01 and 0.06 indicate a small effect, values between 0.06 and 0.14 indicate a medium effect, and values above 0.14 indicate a large effect (Cohen, 1988). Two 2×3-rmANOVAs for each gait parameter with within-factors *Walking Condition* (unloaded walking, loaded walking) and *Task Condition* (no secondary task, STR, SUB) were conducted to test differences in CV and mean values. The statistical analysis was performed using SPSS Statistics 28.0 (IBM, Armonk, NY, United States).

## 3 Results

### 3.1 Variability of gait parameters

The 2 × 3-rmANOVA revealed that there was not a statistically significant interaction between *Walking Condition* and *Task Condition* for all gait variability parameters: Step length (*F* [1.31, 18.21] = 1.37, *p* = 0.268, $\eta_{p}^{2}$ = 0.09), step width (*F* [2, 28] = 1.19, *p* = 0.319, $\eta_{p}^{2}$ = 0.08), stride time (*F* [2, 28] = 0.51, *p* = 0.608, $\eta_{p}^{2}$ = 0.04) and double support time (*F* [2, 28] = 1.03, *p* = 0.369, $\eta_{p}^{2}$ = 0.07). It is important to note, that in this case step length variability violated normal distribution and sphericity assumption.

**Figure 4:**
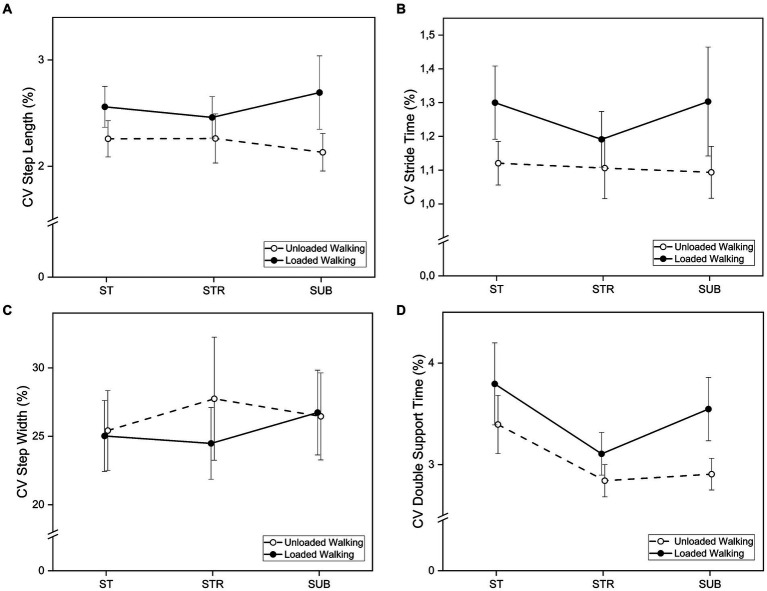
Coefficients of variation (CV) of **(A)** step length, **(B)** stride time, **(C)** step width, and **(D)** double support time for loaded and unloaded walking during single task walking (ST), dual-task walking with the Stroop test (STR) and dual-task walking with the subtraction task (SUB). Bars indicate standard errors.

A significant main effect of *Walking Condition* on CV of step length (*F* [1, 14] = 5.67, *p* < 0.032, $\eta_{p}^{2}$ = 0.29) was found. Step length variability increased during loaded walking (see Figure 4; Table 2). No significant main effects of *Walking Condition* were found for stride time (*F* [1, 14] = 4.06, *p* < 0.064, $\eta_{p}^{2}$ = 0.23), double support time (*F* [1, 14] = 2.83, *p* = 0.115, $\eta_{p}^{2}$ = 0.17) and step width (*F* [1, 14] = 0.12, *p* = 0.736, $\eta_{p}^{2}$ < 0.01). However, variability in stride time and double support time showed statistical trends that also indicate an increase in variability during loaded walking, supporting hypothesis H1b.

**Table 2:** Coefficients of variation and mean values of gait parameters for each experimental condition.

	Loaded walking			Unloaded walking		
	Single task	Dual-task (STR)	Dual-task (SUB)	Single task	Dual-task (STR)	Dual-task (SUB)
**CV**						
Step length (%)	2.6 (0.7)	2.5 (0.8)	2.7 (1.3)	2.3 (0.7)	2.3 (0.9)	2.1 (0.7)
Step width (%)	25.0 (10.0)	24.5 (10.1)	26.7 (12.0)	25.4 (11.3)	27.7 (17.4)	26.5 (12.3)
Stride time (%)	1.3 (0.4)	1.2 (0.3)	1.3 (0.6)	1.1 (0.2)	1.1 (0.4)	1.1 (0.3)
Double support time (%)	3.8 (1.6)	3.1 (0.8)	3.5 (1.2)	3.4 (1.1)	2.8 (0.6)	2.9 (0.6)
**Mean values**						
Step length (mm)	631 (37)	632 (38)	631 (35)	604 (4.1)	603 (43)	604 (41)
Step width (mm)	102 (28)	101 (27)	100 (27)	87 (2.4)	90 (26)	92 (27)
Stride time (ms)	1,208 (83)	1,203 (65)	1,201 (63)	1,147 (67)	1,142 (57)	1,148 (54)
Double support time (ms)	356 (10)	354 (9)	354 (8)	377 (9)	372 (8)	373 (8)

Values represent group mean (standard deviation). STR, Stroop-Test; SUB, Subtraction-Task; CV, Coefficient of Variation.

There was a significant main effect of *Task Condition* on double support time variability (*F* [2, 28] = 4.31, *p* = 0.023, $\eta_{p}^{2}$ = 0.24). Bonferroni-corrected *post-hoc* analysis revealed a statistical trend for differences from single task (ST) to STR (MDiff = 0.62, 95%-CI [−0.03, 1.28], *p* = 0.066). Inspection of Figure 4 suggests that variability in double support time is lower when walking while performing the STR task compared to single task walking. As hypothesized (H1a) variability increased again during SUB for loaded, but not unloaded walking, leading to a U-shaped curve. Variability in double support time was not significantly different from ST to SUB (MDiff = 0.37, 95%-CI [−0.29, 1.03], *p* = 0.455) and from STR to SUB (MDiff = −0.25, 95%-CI [−0.62, 0.12], *p* = 0.255). There were no significant main effects of *Task Condition* on CV of step length (*F* [2, 28] = 0.06, *p* = 0.943, $\eta_{p}^{2}$ < 0.01), step width (*F* [2, 28] = 0.74, *p* = 0.486, $\eta_{p}^{2}$ = 0.05) and step time (*F* [2, 28] = 0.35, *p* = 0.707, $\eta_{p}^{2}$ = 0.02).

### 3.2 Mean values of gait parameters

The interaction between *Walking Condition* and *Task Condition* was not significant for all mean gait parameters: Step length (*F* [2, 28] = 0.39, *p* = 0.683, $\eta_{p}^{2}$ = 0.03), step width (*F* [1.35, 18.84] = 2.21, *p* = 0.149, $\eta_{p}^{2}$ = 0.14), stride time (*F* [2, 28] = 1.09, *p* = 0.349, $\eta_{p}^{2}$ = 0.07) and double support time (*F* [2, 28] = 0.91, *p* = 414, $\eta_{p}^{2}$ = 0.06).

**Figure 5:**
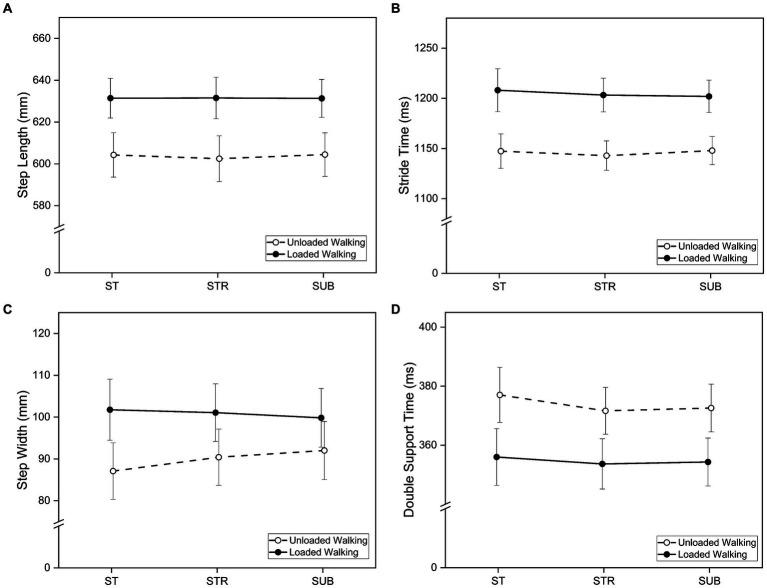
Mean values of **(A)** step length, **(B)** stride time, **(C)** step width, and **(D)** double support time for loaded and unloaded walking during single task walking (ST), dual-task walking with the Stroop-Test (STR) and dual-task walking with the Subtraction-Task (SUB). Bars indicate standard errors.

The 2 × 3-rmANOVA showed significant main effects of *Walking Condition* on mean values of step length (*F* [1, 14] = 98.49, *p* < 0.001, $\eta_{p}^{2}$ = 0.88), stride time (*F* [1, 14] = 65.56, *p* < 0.001, $\eta_{p}^{2}$ = 0.82), step width (*F* [1, 14] = 4.72, *p* = 0.047, $\eta_{p}^{2}$ = 0.25) and double support time (*F* [1, 14] = 30.60, *p* < 0.001, $\eta_{p}^{2}$ = 0.69). In line with hypothesis H2a, loaded walking resulted in increased step length, step width, and stride time, while decreasing double support time compared to unloaded walking (see Figure 5; Table 2).

There were no significant main effects of *Task Condition* on mean values of step length (*F* [1.40, 19.53] = 0.06, *p* = 0.888, $\eta_{p}^{2}$ < 0.01), step width (*F* [2, 28] = 0.29, *p* = 0.754, $\eta_{p}^{2}$ = 0.02), stride time (*F* [1.29, 18.01] = 0.27, *p* = 0.670, $\eta_{p}^{2}$ = 0.02) and double support time (*F* [1.26, 17.68] = 1.45, *p* = 0.251, $\eta_{p}^{2}$ = 0.09). This means that hypothesis H2b cannot be confirmed.

## 4 Discussion

This study aimed to examine the effects of modified leg mechanics on cognitive-motor interference in dual-task walking. For this purpose, a within-subject experimental design was employed. Participants walked on a treadmill with and without weight cuffs bilaterally attached to their thighs and shanks under different cognitive demand levels. The study investigated the effects of physical load (weight cuffs) and cognitive load (secondary tasks) on variability, as a measure of motor control, and mean values of step length, stride time, step width and double support time. The study found that walking with modified leg mechanics in challenging dual-task situations could lead to an increase in cognitive-motor interference. Additionally, participants seemed to prioritize maintaining their posture over the cognitive task in challenging dual-task situations during walking with modified leg mechanics.

### 4.1 Dual-process account observed for double support time variability during loaded walking

Literature supports a dual-process account in dual-task walking (Lövdén et al., 2008; Verrel et al., 2009; Decker et al., 2016). Accordingly, simple secondary tasks seem to promote an external focus of attention resulting in reduced gait variability compared to single-task walking. Conversely, complex secondary tasks lead to a competition for cognitive resources, potentially resulting in increased gait variability (U-shaped relationship). The model also proposes that the interplay between external focus and resource competition is influenced by age. The hypothesis was that modified leg mechanics demands the postural reserve to a similar extent as aging. In the conducted study, participants rated the Stroop test (STR) as less mentally demanding than the subtraction task (SUB) (Riedel et al., 2023). Consequently, it was anticipated that variability would decrease for the STR task (which involves an external focus of attention) and increase for the SUB task (where cognitive resource competition occurs) during loaded walking (H1a).

However, this was solely evident in the case of double support time (DST) variability (see Figure 4). The other variability parameters showed a similar pattern resembling a U-shaped curve, yet lacked significant effects. DST variability decreased from single task walking to walking while concurrently performing the STR task, both for unloaded and loaded walking, indicating an externalized focus of attention. Conversely, in the more cognitively demanding SUB task, DST variability continued to be lower for unloaded walking, but increased for loaded walking. This observation suggests that competition for cognitive resources outweighs the benefits of an external attentional focus when walking with weight cuffs in cognitively demanding situations. Lövdén et al. (2008) observed that the beneficial effect of an external focus of attention persists among young, healthy adults even when confronted with complex secondary tasks. However, this did not hold true for older adults. Decker et al. (2016) reported similar results regarding step length and step time. For step width, they identified a U-shaped pattern in both younger and older adults. This implies that increased cognitive demand influences balance control in individuals of all ages, while affecting the regulation of rhythmic step patterns exclusively in older adults. Like step width, DST is related to balance control (Gabell and Nayak, 1984). Therefore, the results of the present study imply that increased cognitive demands during loaded dual-task walking primarily impact balance control in healthy young adults. However, step width did not show the expected pattern.

Regardless of the cognitive demand, variability in step length was higher during loaded walking than during unloaded walking. This supports hypothesis H1b. Modified leg mechanics seem to influence gait variability to a similar extent as age-related effects. For instance, Decker et al. (2016) reported that older adults demonstrated increased variability in step length and step time than younger adults.

### 4.2 Mental tracking rather than inhibitory control interferes with motor control

The results also suggest that mental tracking, rather than inhibitory control, may interfere with motor control during loaded walking. This is in line with the findings of Kao and Pierro (2022). Tasks requiring memorization of information while simultaneously engaging in internal mental processes, such as serial subtraction, have a greater interference effect on gait performance. Such mental tracking tasks are suggested to engage neural networks that overlap with those involved in locomotion (Al-Yahya et al., 2011). Specifically, the prefrontal cortex has been implicated in both locomotion and dual-tasking (Holtzer et al., 2011; Hamacher et al., 2015). However, contrasting findings exist. Serial subtraction did not consistently yield the greatest cognitive-motor interferences in studies involving different cognitive tasks (Patel et al., 2014; De Bartolo et al., 2021). Patel et al. (2014) observed that performing a Stroop test resulted in more interference with walking at preferred speed compared to a serial subtraction task, a word list generation task, and a simple reaction time task. Zoccatelli et al. (2010) demonstrated that the Stroop test activates several brain regions, including the dorsolateral prefrontal cortex and the anterior cingulate cortex. According to Patel et al. (2014), the neural activation pattern in the Stroop test indicates that it requires more processing resources than the other tasks studied. This contrasts with the subjective ratings of the cognitive demands of the participants in the present study. Explanations for the differing findings could be task-specific factors. The SUB task in this study, involving the subtraction of sevens from a random three digit number, may have been more complex than in the study by Patel et al. (2014), requiring more cognitive resources. Furthermore, in the visual-verbal STR task, participants had to focus on the monitor in front of the treadmill on which the stimulus was presented, while in the SUB task, participants had the flexibility to shift their visual focus away from the monitor despite instructions to the contrary. To focus on the letters of the stimuli in the STR task participants had to stabilize themselves potentially leading to reduced variability. This aligns with the Supra Postural Task Model (Stoffregen et al., 2000), which could offer an alternative explanation for the reduced variability during dual-task walking.

### 4.3 Shift to a posture first strategy during demanding dual-task situations

From the perspective of the Task Prioritization Framework proposed by Yogev-Seligmann et al. (2012), the shift of attention to the cognitive task (external focus) can be interpreted as the adoption of a “posture second” strategy. However, during loaded walking while performing the SUB task the positive impact of an external focus was nullified. This suggests a reallocation of attentional resources toward the motor task, indicating a shift toward a “posture first” strategy. Such a strategy is adopted when hazards are detected, aiming to stabilize walking and to prevent injuries (Shumway-Cook et al., 1997). Nonetheless, the extent of perceived postural threat influences the amount of attentional resources allocated to the motor task (Yogev-Seligmann et al., 2012). Riedel et al. (2023) reported that during loaded walking, participants who initiated with loaded walking experienced a substantial reduction in cognitive performance for the SUB task (i.e., fewer subtractions per period). In contrast, those who initiated with unloaded walking showed a slight improvement in cognitive performance during loaded walking. Starting with the dual-task conditions involving the simple motor task (unloaded walking) allowed participants some time to become familiar with the dual-task conditions involving the challenging motor task (loaded walking). It appears that these participants perceived the postural threat as less severe compared to those who initiated with loaded walking. Consequently, they allocated fewer attentional resources to the motor task. For both participant groups the shift toward a “posture first” strategy was successful, as evidenced by the fact that DST variability did not surpass the levels observed in the single-task condition. Decker et al. (2016) and Lövdén et al. (2008) reported similar observations. However, it seems that in contrast to the participants who initiated with unloaded walking, the participants who initiated with loaded walking did not have sufficient cognitive resources left to perform the SUB task, which led to the observed cognitive performance decrements (Riedel et al., 2023). Consistent with this, prior research has shown that when confronted with more complex motor tasks, such as responding to unexpected perturbations (Mersmann et al., 2013) or adapting to asymmetric split-belt conditions (Hinton et al., 2020), healthy young adults prioritize maintaining their posture as their primary strategy.

Interestingly, this pattern was not observed during unloaded walking, suggesting that modifications in leg mechanics increase the postural threat and demand the postural reserve of healthy young adults. However, the results also suggest that familiarization alters the perceived threat and thus the amount of cognitive resources allocated to the motor task. Further studies are needed to investigate the time for familiarization with modified leg mechanics or powered exoskeletons, which is crucial for the design of effective training interventions.

### 4.4 Gait pattern influenced by modified leg mechanics but not secondary tasks

Weight cuffs attached to the legs not only increase metabolic rate (Royer and Martin, 2005) but also shift the center of mass of the leg segments and change the overall moment of inertia of the leg (Browning et al., 2007). In this study, participants showed increased step length, step width, and stride time, while double support time decreased, which provides support for hypothesis H2a. However, the present findings challenge the established paradigm of the inverted pendulum hypothesis, which posits that during the stance phase of gait, the human body exhibits behavior analogous to an inverted pendulum, with the body’s center of mass oscillating over the supporting foot akin to a pendulum. This hypothesis suggests that alterations in gait primarily arise from variations in segment length rather than changes in mass. For example, Iosa et al. (2016) demonstrated significant modification of the gait ratio through artificial extension of the lower leg segment, while the addition of 1 kg weights to the lower legs did not yield significant modifications. Similarly to the present study, Browning et al. (2007) observed an increase in stride length and swing time when using foot weights of 4 kg and 8 kg. These weights likely affected the center of mass and inertia properties of body segments, potentially contributing to the observed changes in gait pattern, which are expected to occur mainly during the swing phase. Due to the changed moment of inertia, the leg swings further forward, resulting in an extended step length and stride time. The double support time decreased, indicating an extended swing time and single-leg stance, which could pose higher demands on balance control. A wider step width was presumably adopted to increase the base of support as a possible stabilizing strategy. However, it is also conceivable that the weight cuffs imposed physical constraints on the participants, causing them to increase their step width. Browning et al. (2007) also suggest a shift in strategy with higher weights, prioritizing foot control over energy conservation, as a possible explanation. This implies a reevaluation of the mechanisms underlying locomotor control under varying loads or modified leg mechanics, as it may be the case when wearing an exoskeleton. Jin et al. (2017) reported similar effects. The authors investigated the impact of added masses on walking using pelvic, thigh, and shank cuffs. They found that the weight and inertia of an exoskeleton led to an increased step length, a reduction in step height, and a decreased maximum knee flexion. A comparison of the different loading conditions was made between normal walking and walking with a powered lower limb exoskeleton, revealing that active support could only partially restore normal walking parameters. Specifically, gait parameters primarily affected by inertia such as step length could not be restored.

Contrary to the hypothesis H2b, increased cognitive load did not affect mean gait parameters, aligning with findings from Szturm et al. (2013). In a study involving dual-task treadmill walking, the mean values for step time, swing time, and double support time showed no statistically significant differences between single-task and dual-task walking conditions. However, variability measures increased during dual-task walking. It is plausible that in the context of treadmill-based dual-task walking, the mechanical support provided by the treadmill serves as a regulating mechanism, ensuring the stability of the gait pattern (Wrightson et al., 2020).

### 4.5 Limitations

Effects due to increased cognitive demand often showed no significance in gait parameters. It is possible that the postural reserve of healthy young adults is high enough so that only a small part of attention needs to be allocated to the motor task, even during loaded walking. This could be promoted by the chosen methodology as studies found that treadmill walking increases automaticity (Baek et al., 2023) and enhances cognitive performance (Penati et al., 2020) compared to overground walking. Thumm et al. (2018) reported that in people with Parkinson’s disease, prefrontal cortex activity was lower when walking on a treadmill than when walking on the ground. Therefore, the generalizability of the results on overground walking is limited (Wrightson et al., 2020). By keeping the gait velocity constant, the consistent strategy of reducing gait velocity during dual-task overground walking (Al-Yahya et al., 2011) was prevented. However, it is essential to note that treadmill walking facilitates the collection of a larger number of steps, thus improving the reliability of variability parameters—a well-documented issue in clinical research (Hollman et al., 2010). In this study, 30 strides per condition were analyzed and most parameters showed at least moderate to excellent reliability, while only two parameters showed poor to good reliability (see Table 1). Furthermore, to ensure reliable assessment of performance in the secondary tasks, this study necessitated continuous walking, which would not have been feasible with short overground trials of 5–10 m. However, future studies should prioritize overground walking studies to ensure the transferability of the results to real life. Furthermore, it is important to investigate normalized gait parameters to enhance comparability between studies. Additionally, the secondary tasks used in this study demand different cognitive functions, such as mental tracking in the SUB task and inhibitory control in the STR task (Bayot et al., 2018). For future investigations into cognitive-motor interference, it is recommended to employ secondary tasks that allow for parametric manipulation of cognitive demands, such as the n-back task (Conway et al., 2005). The n-back task requires participants to determine if each stimulus in a sequence matches the one that appeared n items before. As n increases, the task becomes progressively more challenging. The general task remains unchanged, ensuring consistent assessment of the same cognitive function. Another limitation to consider is the moderate sample size (n = 15) in this study. Individual outliers caused greater and overlapping variance in the data, as demonstrated in Figure 4. However, these outliers were not excluded from the analysis, as the extreme values were not caused by measurement errors but rather corresponded to the natural behavior of these individuals.

## 5 Conclusion

The findings of the study show that walking with modified leg mechanics could increase cognitive-motor interference for healthy young adults in challenging dual-task situations. In challenging dual-task situations, effects of cognitive resource competition outweighed the benefits of an external attentional focus during walking with modified leg mechanics. Interestingly, this pattern was not observed during normal walking, suggesting that modifications in leg mechanics increase the postural threat and demand the postural reserve of healthy young adults. However, the results also suggest that familiarization can alter the perceived threat and thus the amount of cognitive resources allocated to the motor task. In this study, physical effort and biomechanics were passively manipulated using weight cuffs, with both factors known to affect automated walking (Clark, 2015). In contrast to the passive weight cuffs, powered lower limb exoskeletons could compensate for the physical effort (Jin et al., 2017), but controlling the exoskeleton can add cognitive demands and thus deteriorate automated walking. The methodology employed in this study can serve as a conceptual framework for exploring the mechanisms underlying cognitive-motor interference in the domain of human-exoskeleton interaction.

## Data availability statement

The raw data supporting the conclusions of this article will be made available by the authors, without undue reservation.

## Ethics statement

The studies involving humans were approved by the Ethics Committee of the Karlsruhe Institute of Technology, Karlsruhe, Germany. The studies were conducted in accordance with the local legislation and institutional requirements. The participants provided their written informed consent to participate in this study. Written informed consent was obtained from the individual(s) for the publication of any potentially identifiable images or data included in this article.

## Author contributions

NR: Conceptualization, Data curation, Formal analysis, Investigation, Methodology, Software, Visualization, Writing – original draft. MH: Conceptualization, Investigation, Methodology, Writing – review & editing. TS: Conceptualization, Funding acquisition, Project administration, Resources, Supervision, Validation, Writing – review & editing. BD: Conceptualization, Funding acquisition, Project administration, Resources, Supervision, Validation, Writing – review & editing.
